# Community-based epidemiological study on hyperuricemia and gout over 5 years in Huang-pu district, Shanghai

**DOI:** 10.1186/ar3627

**Published:** 2012-02-09

**Authors:** Hui Du, Shun-Le Chen, Chun-De Bao, Xiao-Dong Wang, Yuan Wang, Yue-Ying Gu, Kusuki Nishioka

**Affiliations:** 1 Department of Rheumatology, Ren Ji Hospital, Shanghai Jiaotong University School of Medicine, Shanghai 200001, China; 2 Institute of Medical Science, Tokyo Medical University, Tokyo 160-8402, Japan

## Background

A community-based survey on the prevalence of hyperuricemia and associated factors was carried out in 1996 and 2001.

## Materials and methods

In the target community in1996, 2037 dwellers (age≥15 years old) were interviewed with relevant questionnaires from house to house. According to even house number, 807 blood samples (age≥ 40 years old) were taken for serum uric acid (SUA) levels measured with the uricase-peroxidase enzymatic method. In 2001, 830 residents ≥ 40 years of age were taken for SUA levels measured with the same enzymatic method. Cholesterol, triglyceride, blood urea nitrogen, glycosylated hemoglobin, ESR, rheumatoid factor etc were measured as possible risk factors to enter the multiple logistic regression analysis on hyperuricemia.

## Results

The prevalence of hyperuricemia was 15.1% (51 cases/338, SUA>7 mg/dl) in men, 8.7% in women (41 cases/469, SUA>6 mg/dl) and seven gout male patients were found in 1996. The prevalence of hyperuricemia was 19.5% (52 cases/266, SUA>7 mg/dl) in men, 12.6% (71 cases/564, SUA>6 mg/dl) in women in 2001.The prevalence of gout in 2037 dwellers in Huangpu District was 0.77% in men and 0.34% in both sexes in 1996 [1].

## Conclusions

The mean SUA level in each age group in 2001 was higher than that of in 1996(see Table 1). The prevalence of hyperuricemia was increased rapidly (Male: 15.1% in 1996 to 19.5% in 2001; Female: 8.7% in 1996 to 12.6% in 2001 p < 0.05). Azotemia (≥23 vs. <23 mg/dl), hypertriglyceridemia (≥200 vs. <150 mg/dl, 150--200 vs. <150 mg/dl) were the associated risk factors by multiple logistic regression analyzing the independent effect of each variable on hyperuricemia.

**Table 1:** Comparison of SUA levels in different age group over 5 years

*Year/Age*	*40-49*	*50-59*	*60+*
Male 2001	**5.85 ± 1.02 (56)	*6.04 ± 1.14 (74)	6.20 ± 1.32 (136)
1996	5.38 ± 1.06 (100)	5.53 ± 1.30 (50)	5.90 ± 1.45 (188)
Female 2001	4.19 ± 0.88 (164)	4.72 ± 1.07 (146)	**5.14 ± 1.17 (254)
1996	4.13 ± 0.94 (118)	4.49 ± 1.05 (84)	4.74 ± 1.07 (267)
